# Feasibility and Immediate Effects of Dynamic Exercise Using Mixed Reality for Balance Improvement in Older Adults and Individuals With Neurologic Disorders

**DOI:** 10.1016/j.arrct.2026.100636

**Published:** 2026-05-08

**Authors:** Kazuki Ushizawa, Shintaro Uehara, Akiko Yuasa, Daisuke Matsuura, Yoshitaka Wada, Hirohisa Watanabe, Yohei Otaka

**Affiliations:** a Department of Rehabilitation Medicine, Fujita Health University School of Medicine, Aichi, Japan; b Faculty of Rehabilitation, Fujita Health University School of Health Sciences, Aichi, Japan; c Japan Society for the Promotion of Science, Tokyo, Japan; d Department of Neurology, Fujita Health University School of Medicine, Aichi, Japan

**Keywords:** Aged, Cerebrovascular disorders, Parkinsonian disorders, Rehabilitation, Virtual reality

## Abstract

**Objective:**

To investigate the feasibility and immediate effects of dynamic exercise using mixed reality (MR), a technology that allows real-time interaction between physical surroundings and computer-generated virtual objects, in older adults and individuals with Parkinson’s disease and stroke.

**Design:**

Prospective single-arm intervention study.

**Setting:**

Rehabilitation ward in the hospital.

**Participants:**

Thirty-one participants, including 10 community-dwelling older adults without neurologic or neuromuscular disorders (4 women; mean age, 70.4 y; SD, 3.0), 11 outpatients with Parkinson’s disease (4 women; mean age, 68.4 y; SD, 7.6), and 10 individuals with a stroke (3 women; mean age, 62.8 y; SD, 16.2) in a subacute rehabilitation ward were recruited.

**Interventions:**

Participants performed MR-based exercise for approximately 20 minutes.

**Main Outcome Measures:**

After exercise, participants rated their level of simulator sickness using the Simulator Sickness Questionnaire (SSQ). They further rated their enjoyment, continuation, exercise effectiveness, and fatigue using a Numerical Rating Scale. The Timed Up and Go test and Functional Reach Test were performed both before and after the exercise.

**Results:**

The median SSQ-nausea, SSQ-oculomotor, SSQ-disorientation, and SSQ-total scores were 9.5, 7.6, 0.0, and 7.5, respectively, indicating a low likelihood of simulator sickness. The median Numerical Rating Scale scores for enjoyment, continuation, effectiveness, and fatigue were 8.0, 8.0, 7.5, and 2.0, respectively. The Timed Up and Go test in individuals with stroke and Functional Reach Test in older adults showed significant improvement after exercise (*P*<.05).

**Conclusions:**

Dynamic exercises with MR may be sufficiently feasible to perform balance exercises with less risk of simulator sickness, offering the potential to improve balance ability.

## Highlights

• MR-based exercise is feasible and well-tolerated in older adults and patients.
• Minimal simulator sickness was observed after a single 20-min MR session.
• Participants reported high enjoyment, motivation, and perceived effectiveness.
• Balance improved post-exercise in older adults and individuals with stroke.
• Adjustable, game-like MR tasks may support engagement and safe physical activity.

Declines in balance ability associated with aging and neurologic disorders increase the risk of falls during activities of daily living.^1^ Impaired balance ability arises from multiple factors, including age-related visual changes, decreased proprioception, and muscle weakness in older adults^2^^,^^3^; motor symptoms in individuals with Parkinson’s disease^4^; and muscle weakness and somatosensory deficits after stroke.^5^ Impaired balance ability increases the risk of falls, leading to a restricted life space^6^ and, ultimately, a poor quality of life.^7^ Therefore, rehabilitation training for impaired balance is critical to prevent falls and maintain or improve psychosocial activities.

Therapeutic exercise (particularly dynamic exercise involving weight shifting) has been shown to effectively improve balance.^8^ Such exercises are performed in an unstable environment that requires continuous adaptation. However, in clinical settings, balance exercises are often less dynamic because of environmental and psychological limitations, which restrict movement variability and intensity.

In recent years, virtual reality (VR)-based balance exercise has emerged as a promising approach to address these limitations. By enabling real-time interaction between users and the virtual environment,^9^ VR facilitates rehabilitation interventions that are not feasible in real-world settings.^10^^,^^11^ It supports highly dynamic exercises by presenting tasks across a wide range of directions within a virtual space, with difficulty that can be quantitatively adjusted. VR-based balance exercises improve gait and balance more effectively than conventional exercise.^12^, ^13^, ^14^ However, caution is required, as VR is associated with risks including dizziness, simulator sickness, falls, and ultimately injury.^15^ In particular, immersive VR may induce fear, as users must rely solely on virtual input in the absence of real-world environmental cues.

In this study, we focused on dynamic exercise using mixed reality (MR), in which physical environments and digital information coexist and interact in real time.^16^ MR has several advantages. It provides tasks with virtual objects while maintaining visibility of the real environment. It may also decrease the risk of simulator sickness compared with VR because of its lower level of immersion.^17^ This reduced immersion suggests that MR can deliver dynamic exercises within a safer environment than immersive VR. Accordingly, we aimed to investigate the feasibility and immediate effects of MR-based dynamic exercise in older adults, individuals with Parkinson’s disease, and those with stroke.

## Methods

### Study design and participants

This prospective single-arm intervention study was approved by the Ethics Review Committee of Fujita Health University (approval no. HM22-325) and registered in the UMIN Clinical Trials Registry (UMIN no. UMIN000050452).

Thirty-one participants were enrolled between April 2023 and July 2024, including 10 community-dwelling older adults (4 women; mean age, 70.4 y; SD, 3.0), 11 individuals with Parkinson’s disease (4 women; mean age, 68.4 y; SD, 7.6), and 10 with stroke (3 women; mean age, 62.8 y; SD, 16.2). Older adults were recruited from nonmedical hospital workers and the community-dwelling older adults, who were naïve to the purpose of the study through posters displayed in university and hospital facilities and leaflets distributed by the research team. Participants with Parkinson’s disease and stroke were recruited from the Department of Neurology and the Department of Rehabilitation Medicine at Fujita Health University Hospital. Written informed consent was obtained from all participants before their inclusion in the study, in accordance with the Declaration of Helsinki (1964, as revised in 2013). Older adults aged ≥65 years with no history of brain or neuromuscular disorders and individuals with Parkinson’s disease and stroke (first-ever or recurrent) aged ≥18 years who were able to stand independently with or without an assistive device were included. Meanwhile, individuals with cognitive deficits preventing comprehension of instructions or exercise rules, severe visual impairment, orthopedic conditions or rheumatoid arthritis that interfered with exercise, low back pain or lower limb pain that limited exercise performance, and a vestibular disorder or a history of seizures were excluded.

### Intervention

All participants performed a single 20-minute session of MR-based dynamic exercises. This duration was selected to align with the Japanese health insurance system, where every 20 minutes of rehabilitation service is covered by the insurance. A researcher was beside each participant throughout the session to ensure safety and prevent falls. Participants with Parkinson’s disease performed the exercise in the “on” state, reflecting periods of optimal medication effect.

**Fig 1:**
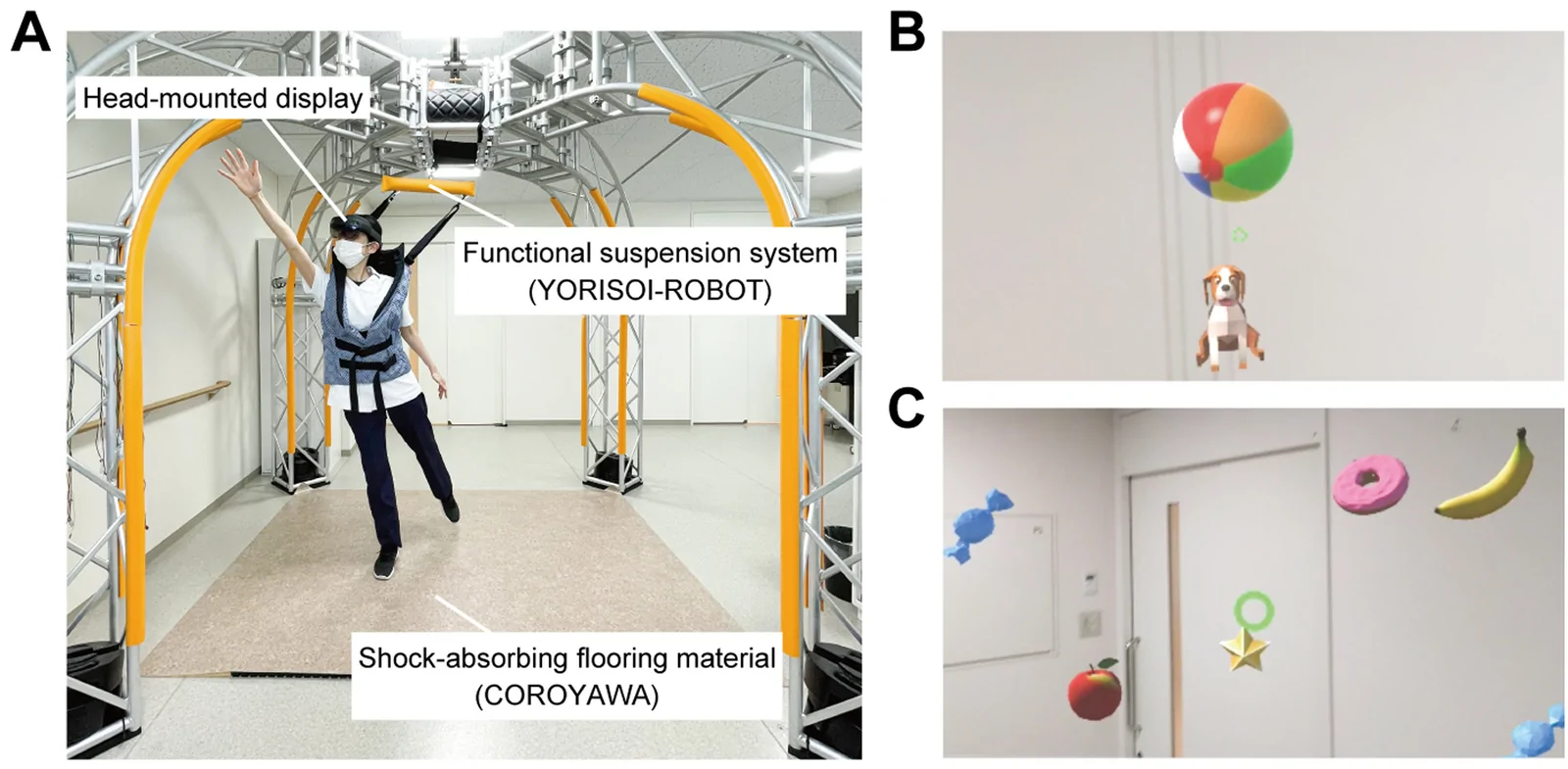
Mixed reality (MR)-based exercise settings. (A) MR-based exercise was performed in a secure environment equipped with a functional suspension system and a shock-absorbing flooring material. Participants performed 2 dynamic exercises: (B) volleyball and (C) object search and touch tasks.

The exercise used an MR application, REHAMARU (Techlico, Inc.),^a^ delivered via a head-mounted display (HoloLens 2; Microsoft Corp.)^b^ (fig 1A). This head-mounted display tracks head, eye, and hand movements using visible light and infrared cameras. Two tasks from REHAMARU^a^ were selected (fig 1B and C). In the first task (“volleyball task”), participants were instructed to return a virtual ball coming from the front to either side of their body. Successful performance required coordinated left-right stepping and arm reaching. Task difficulty was adjusted on the basis of participant performance. In the volleyball task, the speed of the incoming ball and its distance from the body were increased when the participant successfully returned approximately 70%-80% of the balls. Conversely, when the success rate fell below 70%, the incoming ball slowed, and the location of the incoming ball was moved closer to the participant’s body. In the second task (“object search and touch task”), participants were required to touch virtual objects that appeared in a 360° area around them within a limited time. The virtual objects disappeared upon being touched. Completion of the task required participants to change their facing direction, squat, and reach with their arms. Task difficulty was adjusted by increasing the number of objects and the spatial range in which they appeared whenever the participants successfully touched all objects within a limited time. Each participant performed each task for 60-90 seconds and repeated it multiple times (range, 2-6 times). Notably, 2 of the 31 participants performed only the “volleyball task” on request. After several repetitions of each task, the participants were allowed to choose which task to continue for the remainder of the exercise. Task difficulty was subjectively adjusted by a researcher based on a preliminary assessment, considering the participant’s performance and fatigue.

For safety, MR-based exercises were conducted in combination with 2 devices: YORISOI-ROBOT (Sanyo Homes Corp.)^c^ and COROYAWA (Magic Shields, Inc.)^d^ (fig 1A). The YORISOI-ROBOT^c^ is a functional suspension system designed to mitigate the impact of falls by decelerating the fall when its actuator detects a sudden change in velocity. In addition, the system supports dynamic movements while ensuring safety, allowing a greater range of motion with less restraint compared with a standard harness. The COROYAWA^d^ is a shock-absorbing flooring material that, owing to its mechanical metamaterial structure, provides effective shock absorption to reduce the risk of fractures from falls without interfering with gait and exercise performance.^18^ Flooring (190×220cm) was installed in the exercise area, and MR-based exercises were performed on this surface.

### Outcome measure

#### Feasibility

Participants were asked to assess their level of simulator sickness using the Simulator Sickness Questionnaire (SSQ)^19^ and to provide subjective feedback after completing the MR-based exercises. The SSQ is a validated self-report questionnaire designed to assess the severity of 16 symptoms associated with simulator sickness.^19^ Each symptom was rated on a 4-point scale ranging from 0 (not present) to 3 (severe). The SSQ items can be scored in 2 ways: (1) factor scores are calculated by summing the scores of all relevant items and multiplying by a specific weight: nausea (range, 0-200.34), oculomotor (0-159.18), and disorientation (0-292.32); and (2) the total score is calculated by summing all item scores and multiplying the sum by a specific weight (range, 0-179.52). Levels of enjoyment, perceived potential for continued use, perceived effectiveness, and fatigue were assessed using a Numerical Rating Scale (NRS).^20^ Participants assigned scores ranging from 0 (strongly disagree or no fatigue) to 10 (strongly agree or extreme fatigue) in single-point increments for each item. In addition, the number of adverse events occurring during the exercises was recorded to assess safety.

#### Immediate effect on balance ability

The Timed Up and Go test (TUG)^21^, ^22^, ^23^ and Functional Reach Test (FRT)^24^, ^25^, ^26^ were administered before and after exercise to investigate the immediate effects on balance ability. The participants performed the TUG twice, with the faster trial used as the representative measure. For the FRT, participants completed 3 trials, and the mean of the second and third trials was used as the representative data.

### Statistical analysis

Descriptive statistics, expressed as the medians, were calculated for SSQ and NRS scores across all participants and within each participant group to investigate subjective responses. The median TUG and FRT scores were calculated for all participants and compared pre- and post-exercise using the Wilcoxon signed-rank test to investigate the global effect of MR-based exercise on balance ability. The same analyses were conducted separately within each participant group to investigate potential differences in responsiveness. Effect sizes (*r*) were calculated to quantify the magnitude of the intervention effect. All statistical analyses were performed using SPSS version 26 (IBM Corp.).^e^ Statistical significance was set at *P*<.05.

## Results

### Participants

Participants’ characteristics are provided in table 1. All participants with Parkinson’s disease and stroke were able to walk with or without supervision. None of the participants withdrew from the study, and all completed the 20-minute MR-based dynamic exercise.

**Table 1:** Participants’ characteristics

	Older Adults (n=10)	Parkinson’s Disease (n=11)	Stroke (n=10)
Age, y, means (SD)	70.4 (3.0)	68.4 (7.6)	62.8 (16.2)
Sex, n			
Male	6	7	7
Female	4	4	3
Time since PD diagnosis			
Mean months (SD)	-	60.0 (37.6)	-
Time since stroke			
Mean days (SD)	-	-	68.1 (25.1)
Type of stroke, n			
Infraction	-	-	4
Hemorrhage	-	-	6
Affected side, n			
Left	-	-	7
Right	-	-	3
Walking ability, n			
Without walking aids	10	11	2
Used cane	0	0	3
Used AFO	0	0	1
Used cane and AFO	0	0	4

Abbreviation: PD, Parkinson’s disease; AFO, Ankle-Foot Orthosis.

### Feasibility

A detailed summary of the simulator sickness and NRS scores is presented in table 2. The median SSQ scores among all participants were 9.5 for SSQ-nausea, 7.6 for SSQ-oculomotor, 0.0 for SSQ-disorientation, and 7.5 for the SSQ-total score (supplemental fig S1). Similarly, no signs of severe simulator sickness were observed in any participant group. The median NRS scores for enjoyment, continuation, effectiveness, and fatigue were 8.0, 8.0, 7.5, and 2.0, respectively. No adverse events were reported during the exercise.

**Table 2:** Outcome of simulator sickness and subjective assessment

	Older Adults (n=10)	Parkinson’s Disease (n=11)	Stroke (n=10)
SSQ			
Nausea	9.5 (0.0-19.1)	9.5 (0.0-19.1)	9.5 (0.0-9.5)
Oculomotor	0.0 (0.0-7.6)	7.6 (0.0-30.3)	7.6 (0.0-22.7)
Disorientation	0.0 (0.0-13.9)	0.0 (0.0-55.7)	0.0 (0.0-27.8)
Total Score	5.6 (0.0-7.3)	3.7 (0.0-33.7)	5.6 (0.0-15.0)
NRS			
Enjoyment	9.0 (7.0-10.0)	8.0 (6.0-10.0)	8.0 (5.0-10.0)
Continuation	8.0 (5.0-10.0)	7.0 (5.0-10.0)	8.0 (3.0-10.0)
Effectiveness	8.0 (5.0-10.0)	7.0 (5.0-10.0)	7.0 (6.0-8.0)
Fatigue	2.0 (0.0-6.0)	2.0 (0.0-8.0)	3.5 (0.0-7.0)

NOTE. The medians and ranges are provided. The maximum values for each scale were as follows: 200.34 for SSQ-nausea, 159.18 for SSQ-oculomotor, 292.32 for SSQ-disorientation, 179.52 for SSQ-total score, and 10 for the NRS.

### Immediate effects on balance ability

**Fig 2:**
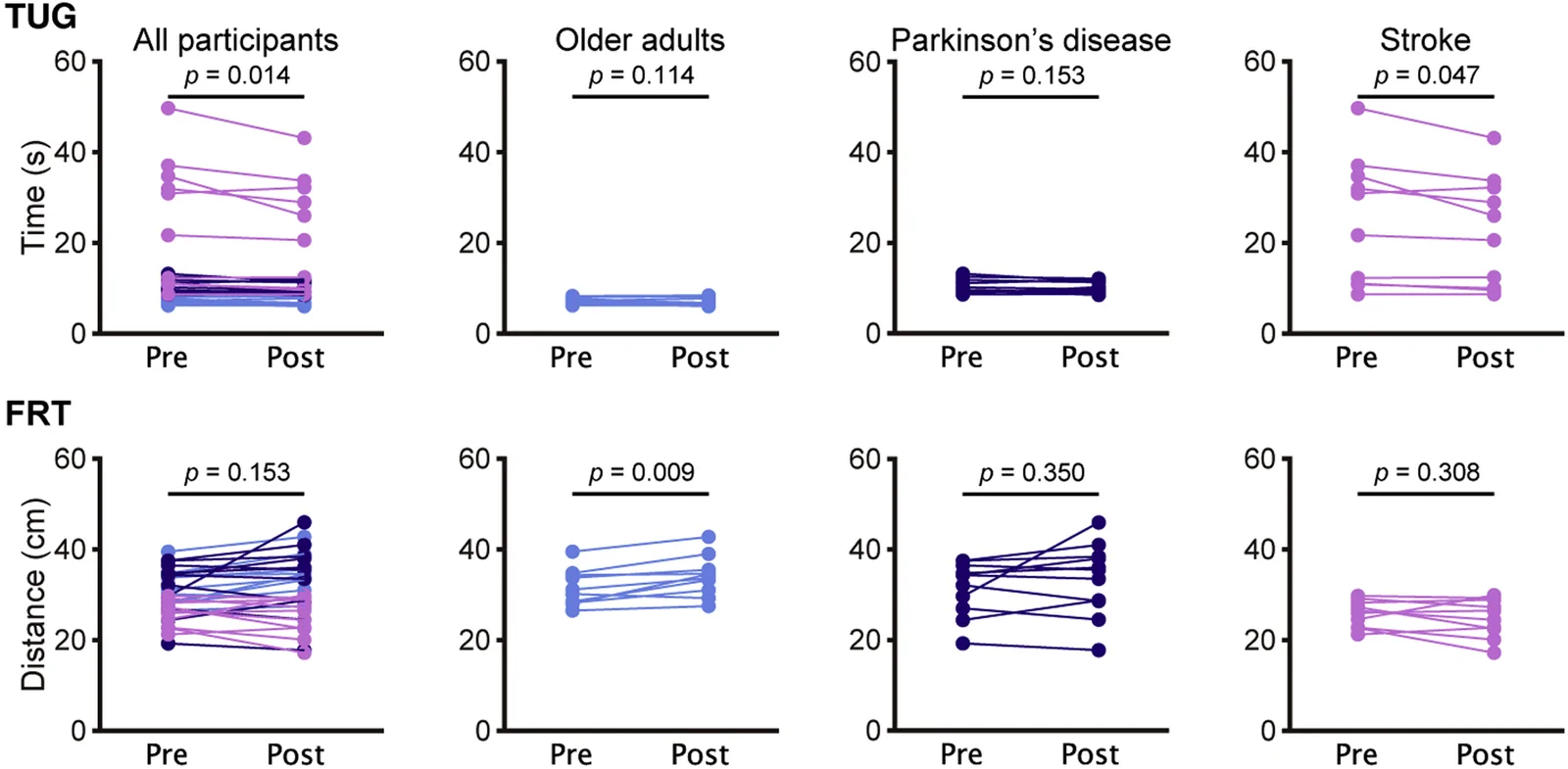
Balance ability outcome before (pre) and after (post) the exercise. Each plot represents individual participants in older adults (light blue), individuals with Parkinson’s disease (dark blue), and those with stroke (pink).

The detailed balance ability outcomes are presented in figure 2. For the TUG, a significant reduction was observed in completion time when all participants were analyzed collectively, decreasing from 14.03 seconds (SD, 10.75) pre-exercise to 13.11 seconds (SD, 9.34) post-exercise (mean difference, 0.92s; 95% confidence interval [CI], 0.15-1.68; *r*=.44; *P*=.014). When analyzed by subgroup, a significant improvement was observed only in individuals with stroke, whose TUG time decreased from 24.88 seconds (SD, 13.30) pre-exercise to 22.53 seconds (SD, 11.47) post-exercise (mean difference, 2.35s; 95% CI, 0.10-4.61; *r*=.63; *P*=.047). By contrast, no significant change was observed in the FRT distance when all participants were analyzed together, with values of 29.69 cm (SD, 5.06) pre-exercise and 30.89 cm (SD, 7.00) post-exercise (mean difference, 1.20 cm; 95% CI, –0.29 to 2.70; *r*=.26; *P*=.153). However, subgroup analysis revealed a significant increase in the FRT distance only among older adults, from 31.49 cm (SD, 3.83) pre-exercise to 34.10 cm (SD, 4.23) post-exercise (mean difference, 2.61 cm; 95% CI, 1.02-4.19; *r*=.82; *P*=.009).

## Discussion

In this study, we investigated the feasibility of a single-session MR-based dynamic exercise and its immediate effects on balance ability in older adults and individuals with Parkinson’s disease and stroke. The findings demonstrated that all participants successfully completed the MR-based exercise without experiencing severe simulator sickness; they also reported a favorable perspective regarding enjoyment, willingness to continue, and perceived effectiveness of the exercise. Furthermore, improvements in the TUG and FRT performance observed in specific groups of participants suggest that MR-based exercise may have the potential to enhance balance ability.

Simulator sickness is a well-recognized adverse effect of VR-based exercise interventions. Here, the median SSQ-total score after the completion of the MR-based exercise was 7.5, indicating the absence of severe simulator sickness. Notably, this value was lower than those reported in previous studies on VR-based rehabilitation.^27^, ^28^, ^29^ This difference may be attributed to the inherent characteristics of MR systems, which overlay virtual elements onto the real-world environment, thereby producing a less immersive experience than VR. Prior research has demonstrated that simulator sickness occurs less frequently in non-immersive VR than in fully immersive VR.^30^^,^^31^ Therefore, the lower level of immersion associated with MR may have contributed to the reduced occurrence of simulator sickness.

A more detailed analysis of simulator sickness symptoms revealed that 66.7% of the 54 responses rated 1 (slight) or 2 (moderate) for “sweating” or “fatigue” (supplemental fig S1). These responses are more likely attributable to physical exertion during dynamic activity rather than to simulator sickness induced by the MR environment. This result supports the assumption that MR-based exercise can facilitate dynamic physical activity while maintaining a low risk of simulator sickness. Although direct evidence is lacking, this effect may have been supported by the safety measures implemented in this study. Given that a high risk of falls can lead to fear of falling and restrict movement, the use of such safety systems may help promote more active and dynamic physical engagement.

The NRS scores for enjoyment, willingness to continue, and perceived effectiveness were high, whereas fatigue levels were low. These findings are consistent with the SSQ results: among the 15 participants who reported fatigue as slight or moderate, 12 (80.0%) rated it as slight. This suggests that the MR-based exercise induced only mild fatigue. These results indicate that the current MR-based exercise can promote physical activity, resulting in modest fatigue while maintaining high levels of participant motivation. A plausible explanation for this favorable perspective is the incorporation of game-like tasks within the MR-based exercise. Gamification is commonly used in VR interventions, as it enhances engagement and motivation.^32^ In this study, the use of virtual objects in game-like tasks likely contributed to the high enjoyment and continuation scores. In addition, task difficulty was adjusted according to individual performance. Tailoring task difficulty to participants’ abilities can positively influence motivation.^33^ One advantage of VR- and MR-based exercise is the ability to quantitatively modulate task difficulty.^11^^,^^28^ The interaction between these 2 factors may have contributed to the highly positive subjective responses to the current exercise.

Improvements in balance ability were observed after the MR-based exercise. Specifically, TUG and FRT performances improved in participants with stroke and older adults, respectively. By contrast, no significant improvements were observed in the TUG performance among older adults and the FRT performance among individuals with stroke. In addition, no significant improvements were detected in either assessment among individuals with Parkinson’s disease. Several factors may explain these differences in immediate effects across participant groups. The absence of TUG improvement among older adults may be attributable to a floor effect. Their mean pre-exercise TUG time was 7.25 seconds, which was faster than the reported mean of 8.1 seconds (95% CI, 7.1-9.0) in the general population of older adults (60-69 y).^34^ This suggests limited potential for further improvement. Differences in movement strategies during the MR-based exercise may also have influenced the FRT outcomes. Based on qualitative observations, older adults performed the tasks using larger, more dynamic steps and pronounced trunk and arm movements. By contrast, individuals with stroke or Parkinson’s disease exhibited smaller step sizes and limited trunk movement. Dynamic balance is closely correlated with trunk function,^35^^,^^36^ and FRT performance has been shown to improve with trunk-involving balance exercises.^37^^,^^38^ Hence, these differences in movement patterns may account for the variations in FRT outcomes. Reported minimal detectable changes are 2.9 seconds for the TUG in individuals with stroke^23^ and 8.2 cm for the FRT in older adults.^39^ Here, the mean improvements after the MR-based exercise were below the previously reported minimal detectable change values. Although these improvements were deemed significant, their clinical relevance may be limited. Further studies are needed to determine optimal exercise parameters to achieve clinically meaningful effects. Several factors may explain the lack of improvement in TUG and FRT performances among individuals with Parkinson’s disease. One possibility is that the frequency and duration of the exercise were insufficient. Balance exercises can improve balance function and mobility, but typically require long-term training (5-12wk).^8^ A dose-response relationship for VR-based exercise has been previously reported,^40^ suggesting that a frequency of 4-7 sessions/wk over 4-7 weeks (totaling >40 sessions) is effective for improving balance function. Accordingly, the lack of improvement in balance among individuals with Parkinson’s disease in the present study may be attributed to the substantially lower exercise dose compared with this threshold. Another potential explanation relates to neuroplasticity. Parkinson’s disease is associated with progressive dopaminergic degeneration in the striatum, leading to reduced synaptic plasticity.^41^ Impairments in synaptic plasticity within the primary motor cortex hinder motor skill learning.^42^ Consistent with this, individuals with Parkinson’s disease exhibit slower learning rates and retention deficits, requiring more practice compared with healthy individuals.^43^ Furthermore, individuals with Parkinson’s disease demonstrate limited generalizability, with skills that are not easily transferred to other tasks, even if those tasks are similar.^44^ These neurobiological characteristics of Parkinson’s disease may have contributed to the lack of improvements in balance observed in our study, particularly in the context of the limited exercise dose.

Notably, as noted earlier, only individuals with Parkinson’s disease did not exhibit significant improvements in balance ability, despite reporting a positive perspective of exercise effectiveness. This highlights a discrepancy between objective and subjective assessments. Such a discrepancy suggests that the exercise may have exerted psychological or motivational benefits, fostering expectations of exercise effectiveness and potentially enhancing motivation and engagement in rehabilitation programs. The enjoyable and engaging nature of MR-based tasks, coupled with adjustable task difficulty, likely contributed to this positive perspective. Whether longer intervention periods could translate these subjective benefits into measurable objective improvements remains an intriguing question for future research.

### Study limitations

This study has some limitations. The participant sample exhibited a sex-related imbalance, which may have introduced bias. Previous studies demonstrated that women are less likely than men to engage in vigorous or skilled physical activities.^45^^,^^46^ Consequently, the high scores for enjoyability, willingness to continue, and perceived effectiveness of the MR-based exercise may reflect this uneven sex-based distribution. In addition, the feasibility and effects of the exercise were assessed in a small sample using a single-arm design with only one exercise session. Further large-scale randomized controlled trials with repeated sessions, longer intervention periods, and control groups performing conventional balance exercises are necessary to confirm the clinical efficacy of MR-based exercises. Finally, the population of individuals with Parkinson’s disease and stroke was limited. Therefore, these findings may not generalize to individuals with Parkinson’s disease and stroke or those with more severe mobility impairments, which should be addressed in future studies.

## Conclusions

MR-based exercises represent a feasible and innovative approach for improving dynamic balance. Future studies are needed to examine the long-term effects of this intervention on dynamic balance.

## Suppliers

a. REHAMARU; Techlico, Inc.
b. HoloLens 2; Microsoft Corp.
c. YORISOI-ROBOT; Sanyo Homes Corp.
d. COROYAWA; Magic Shields, Inc.
e. SPSS, version 26; IBM Corp.

## Disclosure

Techlico, Inc. provided temporary access to the mixed reality application REHAMARU free of charge for the conduct of this study. Techlico, Inc. had no role in the study design, data collection, data analysis, manuscript preparation, or decision to publish.

## Data statement

The data that support the findings of this study are available from the corresponding author.

## Appendix Supplementary materials

Image, application 1

## Footnotes

Supplementary material associated with this article can be found, in the online version, at doi:10.1016/j.arrct.2026.100636.
