# Supplementary material for: Feasibility and Immediate Effects of Dynamic Exercise Using Mixed Reality for Balance Improvement in Older Adults and Individuals With Neurologic Disorders
Source: Arch Rehabil Res Clin Transl. 2026 May 8;8(3):100636. doi: 10.1016/j.arrct.2026.100636 (PMC13591191; doi:10.1016/j.arrct.2026.100636)
Supplement: Supplementary file 1 [file mmc1.docx]

**Supplemental Fig S1** Details of SSQ symptoms. The number of participants for each SSQ symptom is shown.
